# De-Escalation Dual Antiplatelet Therapy Prevail over Potent P2Y12 Inhibitor Monotherapy in Patients with Acute Coronary Syndrome Undergone Percutaneous Coronary Intervention: A Network Meta-Analysis

**DOI:** 10.31083/j.rcm2311360

**Published:** 2022-10-25

**Authors:** Jing-Wen Ding, Yang Chen, Zuo-Zhong Yu, Yuan-Bin Zhao, Kun-Peng Fan, Xiong-Da Yao, Long-Long Hu, Yan-Hui Liao, Tian-Hua Deng, Yi Xia, Han-Hui Liao, Ren-Qiang Yang

**Affiliations:** ^1^Department of Cardiovascular Medicine, the Second Affiliated Hospital of Nanchang University, 330006 Nanchang, Jiangxi, China; ^2^Institute of Cardiovascular Diseases, Nanchang University, 330006 Nanchang, Jiangxi, China

**Keywords:** dual antiplatelet therapy, de-escalation, monotherapy, acute coronary syndrome

## Abstract

**Background:**

Dual antiplatelet therapy (DAPT) with potent P2Y12 inhibitor is the cornerstone of acute coronary syndrome (ACS) management. Balancing the effects of different strategies of antiplatelet therapy including DAPT de-escalation, potent P2Y12 inhibitor monotherapy, and conventional DAPT is a hot topic.

**Methods:**

A systematic search was conducted from the MEDLINE, PubMed, and Embase through October 2021 to identify various DAPT strategies in randomized controlled trials (RCTs) for treatment of ACS patients after undergoing PCI with drug-eluting stent (DES). The network meta-analysis was performed to investigate the net clinic benefit of the DAPT de-escalation, potent P2Y12 inhibitor monotherapy, as well as conventional DAPT. The primary outcome was net adverse clinical events, defined as a composite of major bleeding and cardiac death, myocardial infarction, stroke, stent thrombosis, or target-vessel revascularization. The secondary outcomes include major adverse cardiac events and trial-defined major or minor bleeding.

**Results:**

A total of 14 RCTs with 63,982 patients were included. The DAPT de-escalation was associated with a lower risk of the primary outcome compared with potent P2Y12 inhibitor monotherapy (De-escalation vs monotherapy odds ratio (OR): 0.72 95% confidence interval (CI): 0.55–0.96), and other antiplatelet strategies (De-escalation vs clopidogrel + aspirin OR: 0.49 95% CI: 0.39–0.63; De-escalation vs prasugrel + aspirin OR: 0.76 95% CI: 0.59–0.98; De-escalation vs ticagrelor + aspirin OR: 0.76 95% CI: 0.55–0.90). There were no statistical differences in the incidence of bleeding (DAPT de-escalation vs P2Y12 inhibitor monotherapy OR: 0.73 95% CI: 0.47–1.12) and major adverse cardiac events (DAPT de-escalation vs P2Y12 inhibitor monotherapy OR: 0.79 95% CI: 0.59–1.08) between DAPT de-escalation and potent P2Y12 inhibitor monotherapy.

**Conclusions:**

This network meta-analysis showed that DAPT de-escalation would reduce the net adverse clinical events, compared with potent P2Y12 inhibitor monotherapy, for ACS patients undergone PCI treatment.

## 1. Introduction

Dual antiplatelet therapy (DAPT) combining aspirin and different P2Y12 inhibitors enhances antiplatelet activity and reduces the occurrence of both stent-related and spontaneous myocardial infarction (MI) after acute coronary syndrome (ACS) [1]. European society of cardiology/European association for cardio-thoracic surgery (ESC/EACTS) guidelines suggested that newer generation P2Y12 inhibitors, ticagrelor and prasugrel, were found superior to clopidogrel in decreasing major cardiovascular adverse events (MACEs) rate by reduction of ischemic events [2]. However, other researches have demonstrated that significantly increasing bleeding events were prominently associated with potent antiplatelet therapy [3]. Given the obvious correlation between major bleeding and mortality, there is a strong rationale for assessing the balance of efficacy and safety of antiplatelet therapies in ACS patients especial with high risk of ischemia and bleeding [4, 5, 6].

The TOPIC trial divided the duration of ACS into the early phase of the highest risk of ischemic complications and the chronic phase of the highest risk of hemorrhage events due to potent platelet inhibitors [7]. ACS Patients after percutaneous coronary intervention (PCI) were treated with potent P2Y12 inhibitor plus aspirin, and those without adverse events during the first month were administered to switch to clopidogrel plus aspirin (DAPT de-escalation) or continuation of original treatment (unchanged DAPT). The results revealed DAPT de-escalation is associated with a significant reduction in bleeding events without ischemic events arising. Recently released data from the TALOS-AMI trial suggests DAPT de-escalation strategy was associated with a 45% lower risk of net clinical benefits than the ticagrelor-based DAPT strategy, which was primarily attributed to the reduction in bleeding events [8]. Meanwhile, another randomized clinical trial (RCT) demonstrated that ticagrelor monotherapy after 3 months of DAPT was a promising and potentially optimal antiplatelet strategy over ticagrelor plus aspirin, by inducing a more pronounced reduction of bleeding risk in patients with ACS [9]. Therefore, we conducted a systematic review and network meta-analysis to assess the assets and drawbacks of potent P2Y12 inhibitor monotherapy versus de-escalation strategies in patients with ACS. This approach presents the latest evidence to inform P2Y12 inhibitor choice in ACS patients undergoing PCI.

## 2. Methods

### 2.1 Study Selection and Eligibility Criteria

We conducted this study for comparing the efficacy and safety of different antiplatelet strategies among PCI-treated ACS Patients, including conventional DAPT therapies, DAPT with aspirin plus clopidogrel (C group), DAPT with aspirin and ticagrelor (T group), DAPT with aspirin and prasugrel (P group). DAPT De-escalation was considered as aspirin combined with a potent P2Y12 inhibitor (ticagrelor or prasugrel) switched to aspirin combined clopidogrel or low-dose prasugrel after 1–3 months treatment (D group). As for potent P2Y12 Inhibitor monotherapy, which was considered as maintenance of sole ticagrelor therapy after 1–3 months of ticagrelor combined with aspirin after PCI (M group).

The inclusion criteria for randomized controlled trials (RCTs) were displayed as follows: (1) oral P2Y12 inhibitors were assigned for patients; (2) study population were PCI-treated ACS patients; (3) the relevant cardiovascular and other outcomes were reported (listed in the outcome measures); (4) a follow-up period of at least 3 months; (5) publication language limited to English. The exclusion criteria were also identified as follows: (1) studies not reporting the pre-specified outcomes or reporting unqualified data; (2) those studies with the unreasonable experimental design or animal experiments, case reports; (3) data of non-public publications or conference abstracts; (4) studies focusing on different doses of the same species of P2Y12 agents.

### 2.2 Search Strategy and Data Source

Five authors (JWD, YBZ, KPF, XDY, THD) independently scanned the literature by systematic searches of PubMed, MEDLINE, and EMBASE from inception to October 20, 2021. The searched terms or phrases are summarized as follows: “monotherapy”, “de-escalation”, “ticagrelor”, “clopidogrel”, “prasugrel”, “myocardial infarction”, “acute coronary syndrome”. In addition, we further screened all potentially eligible references from identified articles and pertinent reviews.

### 2.3 Data Extraction and Quality Assessment

Two authors (JWD, YBZ) separately extracted data from eligible studies as follows: type of study, intervention, baseline characters, pre-specified outcomes. The discrepancies of information were resolved through discussion with a third author (KPF). The risk of bias in the selected studies was assessed, based on the Cochrane risk of bias tool for randomized trials. Analogously, two authors (XDY, THD) conducted an assessment and settled the discrepancies by a third author (KPF).

### 2.4 Outcome Measures

The prespecified primary outcome was identified as net adverse clinical events, which is the combination of major bleeding and cardiac death, myocardial infarction, stroke, stent thrombosis or target-vessel revascularization. The major adverse cardiac events (MACEs) included cardiac death, myocardial infarction, stent thrombosis, stroke, or target-vessel revascularization. The bleeding outcome included the occurrence of thrombolysis in myocardial infarction (TIMI) major or minor bleeding; if not available, bleeding academic research consortium (BARC) grade ≥2 bleeding, or PLATO (Platelet Inhibition and Patient Outcomes) major and minor bleeding was defined as substitutes. The major bleeding events were prespecified as TIMI major bleeding; if not available, PLATO major bleeding or BARC grade ≥3 bleeding was defined as substitutes.

### 2.5 Statistical Analysis

We performed statistical analysis of odds ratio (OR), 95% confidence intervals (CI) with the package “mvmeta” of STATA 14.0 (StataCrop, TX, USA) and with RevMan 5.3. (Nordic Cochrane Centre, Denmark) to evaluate heterogeneity across included studies, we computed $I^{2}$ by using Cochrane Q statistic. If $I^{2}$ ranges between 25% and 50%, indicating low heterogeneity, the fixed-effect model would be applied for analysis; high risk of heterogeneity ($I^{2}$ >50%) represents the reason of discrepancy would be identified and the random effect model is used for analysis. Relative Odds Ratio (ROR) was calculated for loop-specific heterogeneity, a *p >* 0.05 indicated qualified consistency between direct and indirect comparison. In addition, the surface under the cumulative ranking (SUCRA) is calculated to hierarchically ranking results by the possibility of being the best, higher SUCRA value represented more pronounced efficacy.

## 3. Results 

### 3.1 Characteristics of Included Studies

A total of 2259 articles were obtained in the initial inspection from databases and manual retrieve. 14 RCTs, with a total of 63,982 patients, were finally included in the qualitative synthesis after eliminating unqualified publications for certain reasons (**Supplementary Fig. 1**).

Table 1 (Ref. [7, 8, 9, 10, 11, 12, 13, 14, 15, 16, 17, 18, 19, 20]) shows the characters of eligible studies. Two trials compared the advancement in therapeutic outcome of prasugrel against clopidogrel [10, 11]. Five studies mentioned comparisons between ticagrelor and clopidogrel among ACS patients undergoing PCI [12, 13, 14, 15, 16]. Three trials assessed the superiority of ticagrelor monotherapy against ticagrelor combined with aspirin in different populations [9, 17, 18]. In the global leaders trial, ticagrelor monotherapy was considered as maintenance of sole P2Y12 inhibitor therapy for 23 months after 1 month of ticagrelor combined with aspirin for PCI-treated patients [17]. As for the TICO trial and TWILIGHT trial, Ticagrelor monotherapy was 3 months of conventional DAPT (ticagrelor plus aspirin), then switched to ticagrelor without aspirin for 12 months among patients who underwent PCI [9, 18]. The discussion about the merits and drawbacks of the DAPT de-escalation occurred in the following studies [7, 8, 19, 20]. The PRAGUE-18 trial defined the DAPT de-escalation therapy as ticagrelor switched to clopidogrel among ACS patients due to economic reasons or drug side effects (most of the conversion occurs within 2 months after PCI) [19]. The TOPIC trial described de-escalation therapy as patients on aspirin and a newer P2Y12 inhibitor (ticagrelor or prasugrel) and without adverse event at 1 month, were assigned to switch to aspirin and clopidogrel for 11 months [7]. The HOST-REDUCE-POLYTECH-ACS trial was administered with prasugrel at 10mg daily in combination with aspirin for 1 month, while the de-escalation group received 5 mg prasugrel and the control group continued to accept 10mg prasugrel [20]. TALOS-AMI trial treated ACS patients with aspirin plus ticagrelor for 1 month, and randomly assigned patients to either the de-escalation of clopidogrel combined with aspirin or the conventional group of continued treatment with ticagrelor combined with aspirin [8].

**Table 1.** **Characteristics of eligible studies**.

Study year	Design	Follow-up (months)	Treatment	Number	Year	Male	DM	Previous stroke	Previous MI	Previous PCI	Previous CABG	STEMI	NSTEMI& UA	PCI (%)
GLOBAL LEADERS 2020 [17]	RCT	24	M	3750	64.6 ± 10.3	76.60%	25.70%	2.60%	23%	32.70%	5.60%	28.30%	71.70%	100%
			T	3737	64.5 ± 10.3	76.90%	24.90%	2.60%	23.60%	32.70%	6.20%	27.50%	72.50%	100%
TICO 2020 [18]	RCT	12	M	1527	61 ± 11	79%	27%	4%	4%	9%	1%	36%	35%	100%
			T	1529	61 ± 11	80%	27%	4%	3%	8%	1%	36%	32%	100%
PLATO 2010 [12]	RCT	12	T	6732	61.0 (53–69)	74.80%	22.70%	3.10%	17.10%	14.10%	5.30%	48.80%	38.20%	100%
			C	6676	61.0 (53–70)	74.70%	23.70%	3.30%	16.90%	13.30%	5.70%	49.50%	37.20%	100%
TWILIGHT 2019 [9]	RCT	15	M	3555	65.2 ± 10.3	76.20%	37.10%	-	28.70%	42.30%	10.20%	35.10%	28.80%	100%
			T	3564	65.1 ± 10.4	76.10%	36.50%	-	28.60%	42.00%	9.80%	34.90%	30.80%	100%
Tang 2016 [13]	RCT	12	T	200	64.36 ± 11.409	71%	29%	16%	8%	-	-	100%	0%	100%
			C	200	64.18 ± 11.088	73%	21%	17%	5%	-	-	100%	0%	100%
PRAGUE-18 2018 [19]	RCT	12	P	630	61.4 (43–78.5)	77.30%	19.80%	-	7.70%	6.90%	1.50%	92%	4.70%	100%
			T	600	61.4 (43–78.5)	77.30%	19.80%	-	7.70%	6.90%	1.50%	92%	4.70%	100%
			D	481	62.3 (44.1–79.3)	73.20%	21.20%	-	9.40%	7.30%	2.10%	92.70%	6.40%	100%
Mohareb 2020 [14]	RCT	12	C	472	47.91 ± 18.1	63.98%	47%	-	-	-	-	25.80%	21.40%	100%
			T	471	49.75 ± 17.84	67.52%	46.20%	-	-	-	-	71.40%	78.50%	100%
TRITON–TIMI 38 2007 [10]	RCT	15	P	6813	61 (53–69)	75%	23%	-	18%	-	8%	26%	74%	99%
			C	6795	61 (53–70)	73%	23%	-	18%	-	7%	26%	74%	99%
ELDERLY ACS 2 2020 [11]	RCT	12	C	524	-	-	-	-	-	-	-	-	-	100%
			P	500	-	-	-	-	-	-	-	-	-	100%
Ren 2016 [15]	RCT	12	C	151	55 ± 8	29.90%	-	-	-	-	-	-	100%	100%
			T	149	56 ± 9.2	31.70%	-	-	-	-	-	-	100%	100%
DISPERSE-2 trial 2007 [16]	RCT	3	C	327	62 ± 11.0	66%	25%	-	28%	17%	11%	0%	100%	100%
			T	334	64 ± 12.1	61%	25%	-	24%	13%	8%	0%	100%	100%
TOPIC 2017 [7]	RCT	12	D	323	60.6 ± 10.2	81%	26%	-	-	-	-	36%	64%	100%
			P/T	323	59.6 ± 10.3	84%	29%	-	-	-	-	43%	57%	100%
HOST-REDUCE-POLYTECH-ACS 2020 [20]	RCT	12	P	1168	58.9 ± 9.1	88.80%	40.90%	1.50%	4.70%	12.70%	-	13.10%	86.90%	100%
			D	1170	58.7 ± 9	89.70%	43.80%	1.20%	3.00%	10.70%	-	14.80%	85.20%	100%
TALOS-AMI 2021 [8]	RCT	12	D	1349	60.1 ± 11.3	83.90%	26.80%	3.90%	-	0%	0.20%	54.40%	45.60%	100%
			T	1348	59.9 ± 11.4	82.40%	27.40%	3.70%	-	0%	0.10%	53.50%	46.50%	100%

*Age is median, median (interquartile range), or mean ± SD; C, clopidogrel + aspirin; P, prasugrel + aspirin; T, ticagrelor + aspirin; M, P2Y12 Inhibitor monotherapy; D, de-escalation; LP, Low-dose prasugrel + aspirin; CABG, coronary artery bypass grafting; DM, diabetes mellitus; MI, myocardial infarction; -, not available; NSTEMI, non-ST-segment elevation myocardial infarction; UA, unstable angina; PCI, percutaneous coronary intervention; STEMI, ST-elevation myocardial infarction; RCT, Randomized clinical trial.

### 3.2 Study Quality

Two authors performed the quality assessment of included studies by the Cochrane Risk of Bias tool [21]. The results are shown in **Supplementary Fig. 2**. Only the jointed endpoint of major adverse cardiac events can be distilled from the PRAGUE -18 trial in certain de-escalation groups, rather than detailed items of MACEs [19]. Mohareb’s article reported every item of adverse events, but it failed to separate those results from diabetic patients, to avoid potential bias of different populations, only the composite endpoint of non-diabetic patients was adopted [14].

### 3.3 Structure of the Network Meta-Analysis

**Fig. 1.**
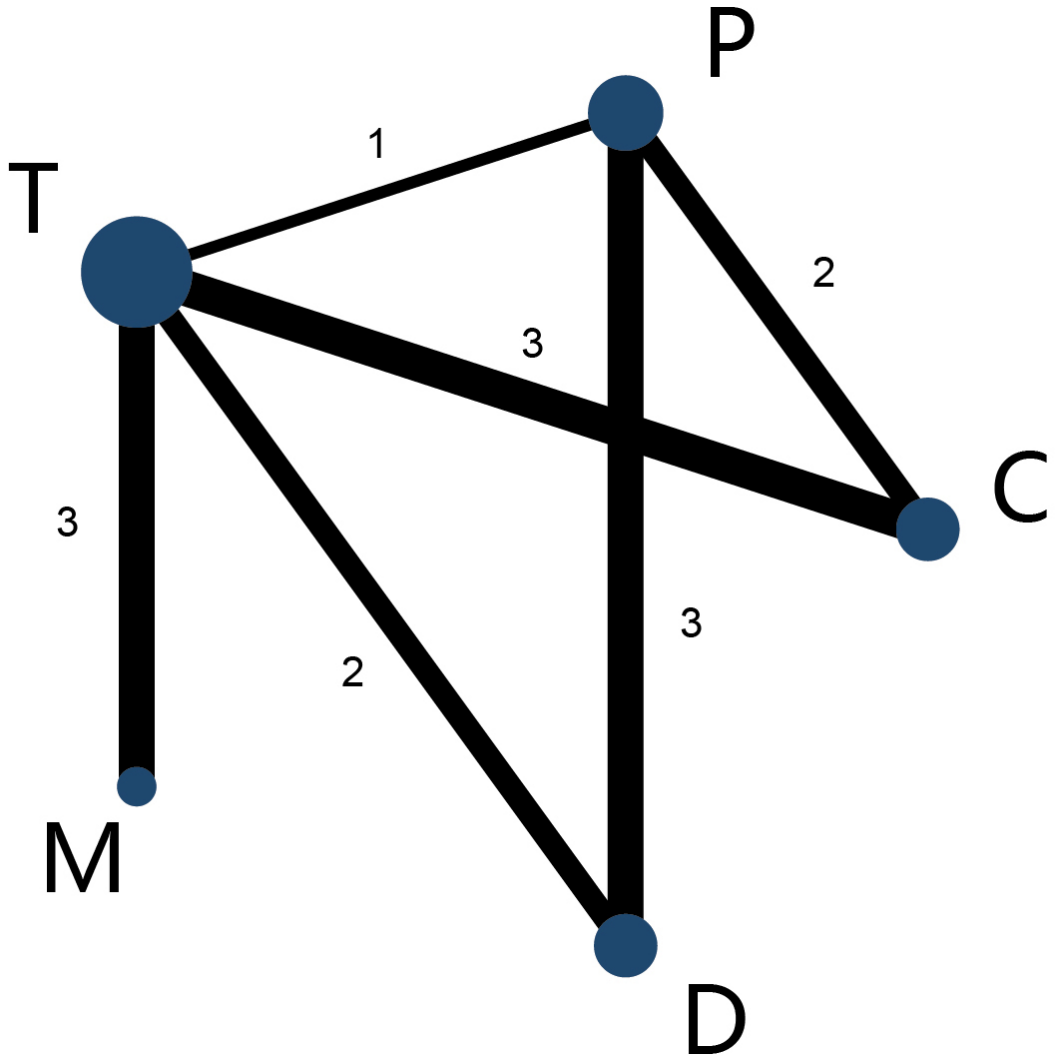
**Network map of interventions of primary outcomes**. The width of the lines connecting 2 strategies reflects the number of patients available for that comparison. The number indicates the number of study arms between the 2 strategies. C, clopidogrel + aspirin; P, prasugrel + aspirin; T, ticagrelor + aspirin; D, de-escalation; MP2Y12 Inhibitor monotherapy.

Five antiplatelet strategies were compared: clopidogrel combined with aspirin (C group), prasugrel combined with aspirin (P group), ticagrelor plus aspirin (T group), ticagrelor monotherapy (M group); DAPT de-escalation (D group). The network plot of the net adverse clinical events for different antiplatelet regiments was constructed in our study (Fig. 1).

### 3.4 Results of Network Meta-Analysis

**Fig. 2.**
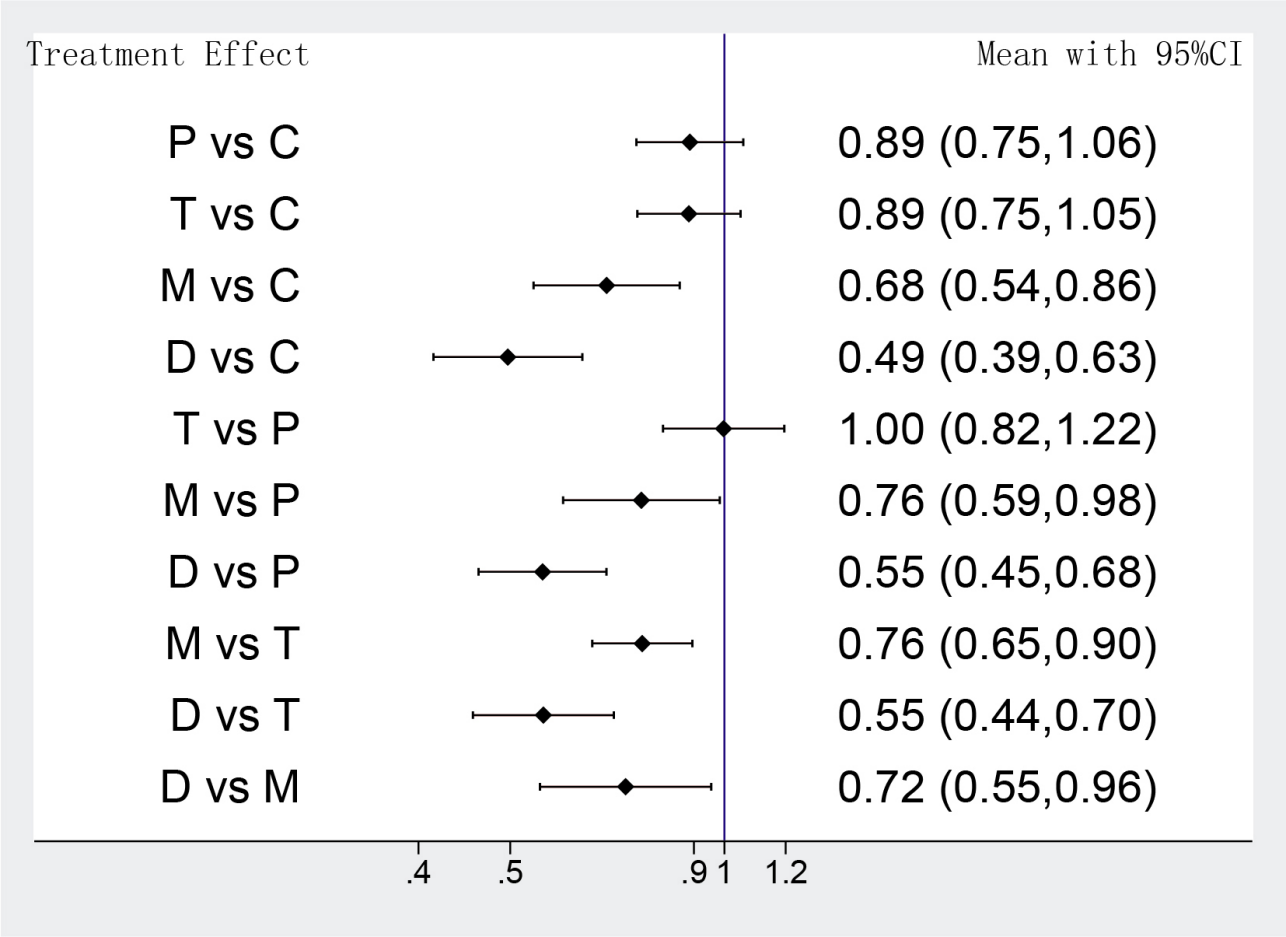
**Network meta-analysis results of primary outcomes**. Values are expressed as OR (95% Confidence Intervals). C, clopidogrel + aspirin; P, prasugrel + aspirin; T, ticagrelor + aspirin; D, de-escalation; M, P2Y12 Inhibitor monotherapy.

The primary outcomes of different strategies were analyzed in 12 RCTs, network meta-analysis showed that DAPT de-escalation therapy took a lower risk in the net adverse clinical events compared with potent P2Y12 monotherapy (D group vs M group OR: 0.72 95% CI: 0.55–0.96), meanwhile, DAPT de-escalation was associated with significantly lower odds in the primary outcomes than conventional DAPT strategies (D group vs C group OR: 0.49 95% CI: 0.39–0. 63; D group vs P group OR:0.55 95% CI: 0.45–0.68; D group vs T group OR: 0.55 95% CI: 0.44–0.70) (Fig. 2); potent P2Y12 monotherapy also showed lower rates in the incidence of net adverse clinical events than potent DAPT groups or clopidogrel plus aspirin (M group vs C group OR: 0.68 95% CI: 0.54–0.86; M group vs T group OR: 0.76 95% CI: 0.65–0.90; M group vs P group OR: 0.76 95% CI: 0.59–0.98). There was no significant difference in the net clinic benefit between potent DAPT groups and clopidogrel-based DAPT (T group vs C group OR: 0.89 95% CI: 0.75–1.05; P group vs C group OR: 0.89 95% CI: 0.75–1.06; T group vs P group OR: 1.0 95% CI: 0.82–1.22) (Fig. 2). Moderate heterogeneity occurred across included studies (*p *= 0.022, $I^{2}$ = 50.6%).

**Fig. 3.**
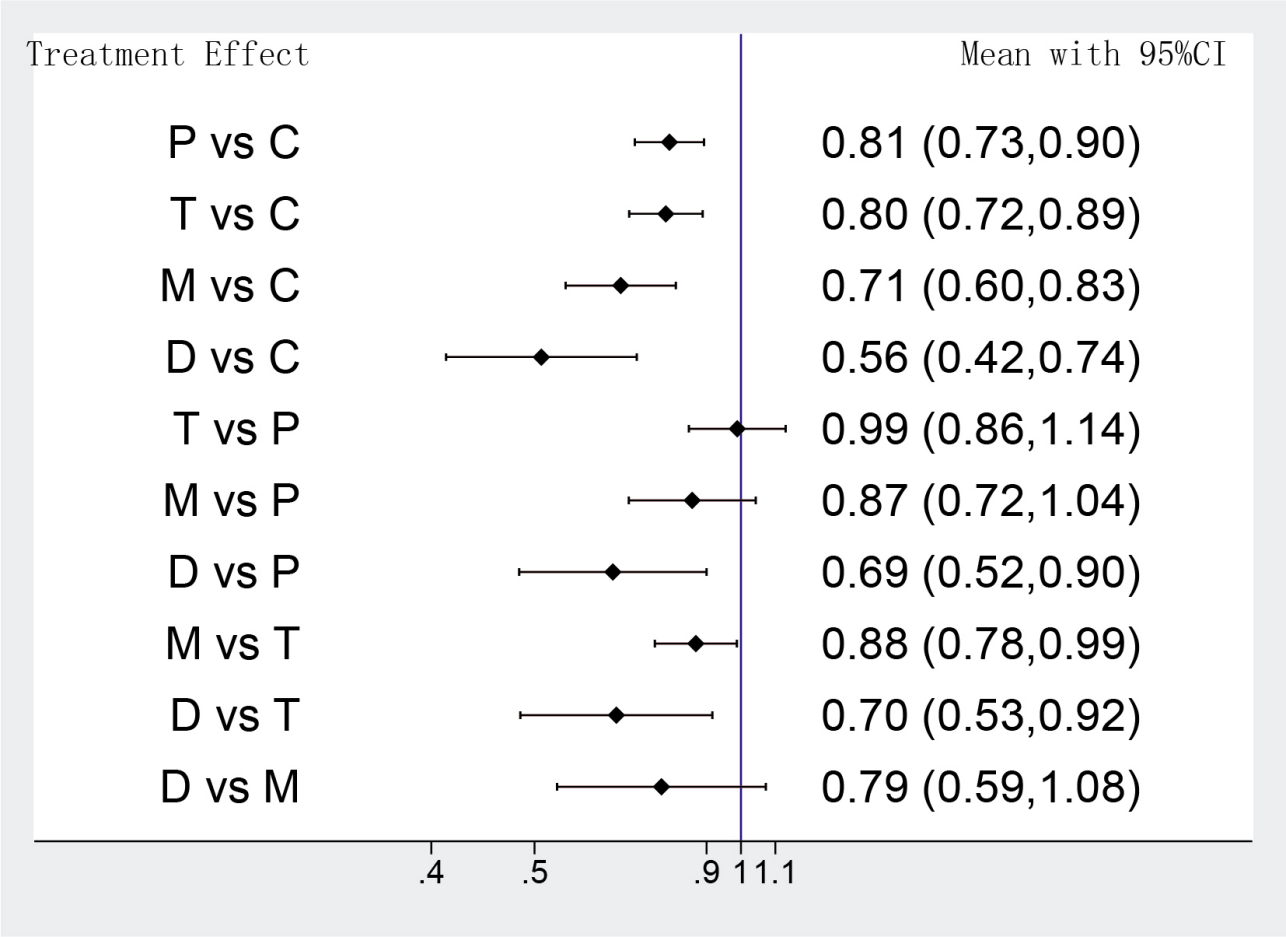
**Network meta-analysis results of MACEs**. Values are expressed as OR (95% Confidence Intervals). C, clopidogrel + aspirin; P, prasugrel + aspirin; T, ticagrelor + aspirin; D, de-escalation; M, P2Y12 Inhibitor monotherapy.

The MACEs of different strategies were analyzed in 14 RCTs, the network meta-analysis showed that DAPT de-escalation therapy took a lower risk in MACEs compared with conventional DAPT groups, but it also indicated that the odds of MACEs were identical between potent P2Y12 monotherapy and DAPT de-escalation (D group vs M group OR: 0.79 95% CI: 0.59–1.08; D group vs C group OR: 0.56 95% CI: 0.42–0.74) (Fig. 3). Furthermore, the benefit in favor of a potent P2Y12 inhibitor monotherapy was significant in the MACEs, compared with clopidogrel-based DAPT (M group vs C group OR: 0.71 95% CI: 0.60–0.83). Rather unexpected, there were slight advantages in low-intensity antiplatelet strategies for reducing MACEs, compared with prasugrel or ticagrelor plus aspirin. (M group vs T group OR: 0.88 95% CI: 0.78–0.99; D group vs P group OR: 0.69 95% CI: 0.52–0.90; D group vs T group OR: 0.70 95% CI: 0.53–0.92). Unsurprisingly, DAPT with a newer P2Y12 inhibitor (prasugrel or ticagrelor) was associated with significantly lower odds of MACEs against clopidogrel plus aspirin (T group vs C group OR: 0.80 95% CI: 0.72–0.89; P group vs C group OR: 0.81 95% CI: 0.73–0.90) (Fig. 3). Heterogeneity was not detectable for comparisons among those trials (*p* = 0.2, $I^{2}$ = 8.8%).

**Fig. 4.**
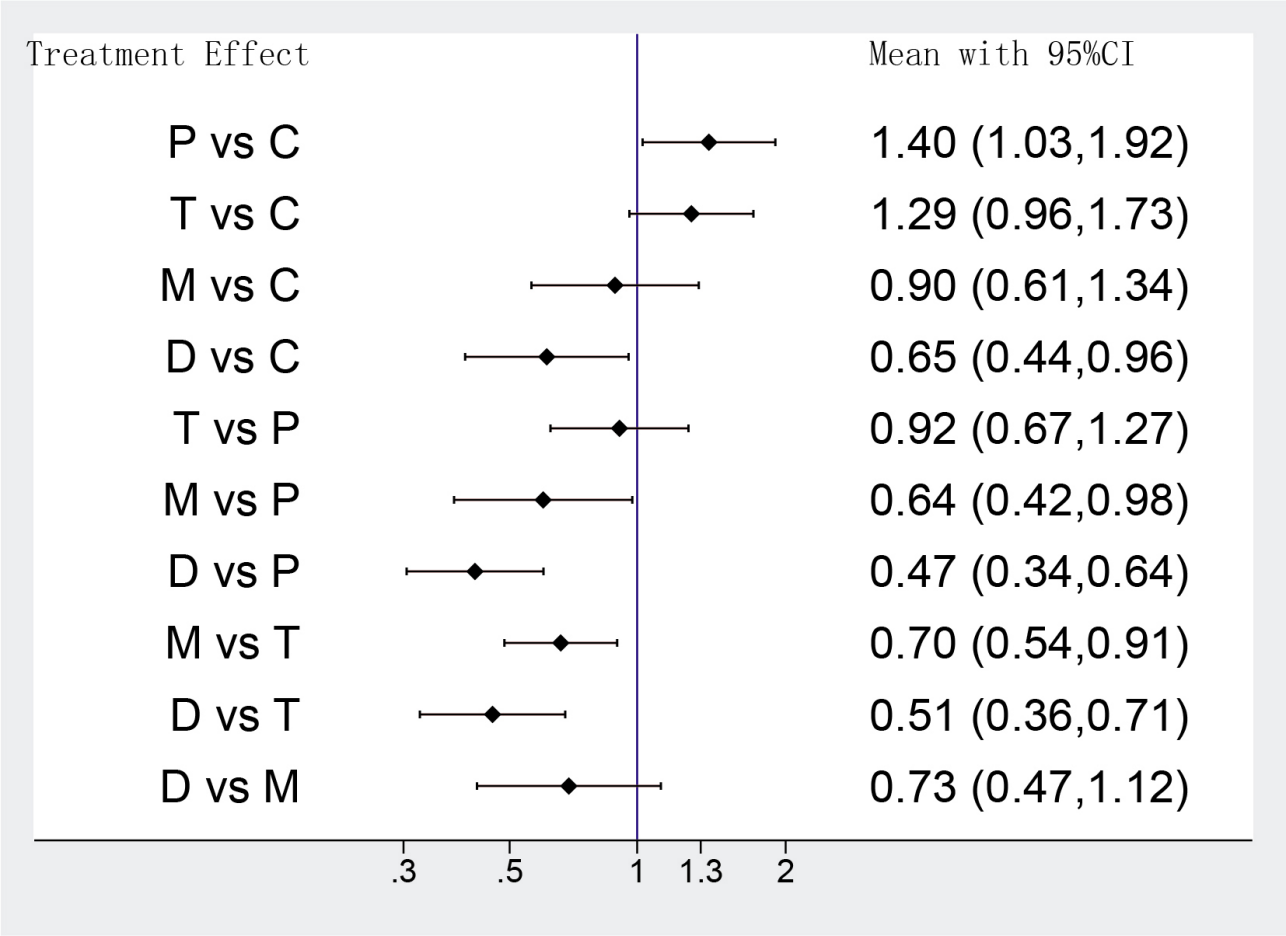
**Network meta-analysis results of bleeding events**. Values are expressed as OR (95% Confidence Intervals). C, clopidogrel + aspirin; P, prasugrel + aspirin; T, ticagrelor + aspirin; D, de-escalation; M, P2Y12 Inhibitor monotherapy.

The analysis of bleeding events was accompanied by moderate heterogeneity among eligible 12 RCTs (*p* = 0.014, $I^{2}$ = 53.6%). The result of the network meta-analysis indicated that there was no significant difference in comparisons between the potent P2Y12 inhibitor monotherapy and DAPT de-escalation in bleeding events (D group vs M group OR: 0.73 95% CI: 0.47–1.12). In addition, DAPT de-escalation was found superior to conventional DAPT (D group vs C group OR: 0.65 95% CI: 0.44–0.96; D group vs P group OR: 0.47 95% CI: 0.34–0.64; D group vs T group OR: 0.51 95% CI: 0.36–0.71). Ticagrelor monotherapy was associated with lower rates of bleeding events compared with potent P2Y12 inhibitors-based DAPT groups (M group vs P group OR: 0.64 95% CI: 0.42–0.98; M group vs T group OR: 0.70 95% CI: 0.54–0.91). As expected, the results suggested most excess bleeding events may be associated with prasugrel or ticagrelor on a background aspirin therapy (P group vs C group OR: 1.40 95% CI: 1.03–1.92; M group vs P group OR: 0.64 95% CI: 0.42–0.98; M group vs T group OR: 0.70 95% CI: 0.54–0.91) (Fig. 4).

The results of the network meta-analysis of 11 RCTs indicated that odds of all-cause death were identical between potent P2Y12 monotherapy and DAPT de-escalation (D group vs M group OR: 1.13 95% CI: 0.60–2.11). P2Y12 inhibitor monotherapy was associated with lower rates of all-cause death compared with clopidogrel or prasugrel-based DAPT (M group vs C group OR: 0.68 95% CI: 0.54–0.86; M group vs P group OR: 0.71 95% CI: 0.54–0.94), and it also showed a tendency towards superiority over ticagrelor-based DAPT without reaching statistical significance (M group vs T group OR: 0.85 95% CI: 0.72–1.00). DAPT de-escalation didn’t show any advantages in lower risk of all-cause death than other treatments. As was expected, ticagrelor plus aspirin was superior to clopidogrel plus aspirin in all-cause death incidence (T group vs C group OR: 0.80 95% CI: 0.69–0.94) (**Supplementary Table 1**). The analysis was accompanied by low heterogeneity (*p* = 0.908, $I^{2}$ = 0%).

11 RCTs were included in the sub-analysis of incidence of MI, with no significant heterogeneity (*p* = 0.837, $I^{2}$ = 0%). There was no significant difference between DAPT de-escalation and potent P2Y12 monotherapy (D group vs M group OR: 0.76 95% CI: 0.41–1.41). DAPT de-escalation and potent P2Y12 monotherapy proved their superior effect in lower the risk of MI over clopidogrel plus aspirin for ACS patients (D group vs C group OR: 0.52 95% CI: 0.29–0.95; M group vs C group OR: 0.69 95% CI: 0.54–0.88). The newer P2Y12 inhibitors (ticagrelor and prasugrel) plus aspirin were also associated with lower rates of MI over clopidogrel plus aspirin (T group vs C group OR: 0.78 95% CI: 0.68–0.90; P group vs C group OR: 0.75 95% CI: 0.66–0.84) (**Supplementary Table 1**).

12 RCTs were included in the sub-analysis of incidence of stroke, without obvious heterogeneity (*p* = 0.432, $I^{2}$ = 1.2%). There were no statistical differences in the incidence of stroke between the 5 antiplatelet strategies (**Supplementary Table 1**).

10 RCTs reported the risk of stent thrombosis, with moderate heterogeneity (*p* = 0.057, $I^{2}$ = 21.2%). There was no significant difference between DAPT de-escalation and potent P2Y12 monotherapy (D group vs M group OR: 0.66 95% CI: 0.17–2.53). In addition, the result showed that potent P2Y12 monotherapy and prasugrel or ticagrelor-based DAPT groups were closely related to the lower incidence of stent thrombosis than clopidogrel combined with aspirin. (M group vs C group OR: 0.59 95% CI: 0.39–0.91; P group vs C group OR: 0.47 95% CI: 0.36–0.62; T group vs C group OR: 0.61 95% CI: 0.45–0.83) (**Supplementary Table 1**).

### 3.5 Ranking of the Treatment Strategies

Among the 5 antiplatelet agents, DAPT de-escalation is considered to be the most effective intervention in terms of inhibiting the net adverse clinical events (**Supplementary Fig. 3**). P2Y12 inhibitor monotherapy ranked best in reducing all-cause mortality (Table 2). Prasugrel plus aspirin was ranked higher in the prevention of stent thrombosis than other treatments (Table 2).

**Table 2.** **Probability ranks with respect to sub-analyses outcomes**.

Treatment		Clopidogrel + aspirin	Prasugrel + aspirin	Ticagrelor + aspirin	Monotherapy	De-escalation
Net adverse clinical events						
	SUCRA	4.9	35.0	35.6	74.8	99.7
	Mean Rank	4.8	3.6	3.6	2	1
Major adverse cardiovascular events						
	SUCRA	0	37.6	39.5	74.8	98.0
	Mean Rank	5	3.5	3.4	2	1.1
Bleeding events						
	SUCRA	56.4	8.6	18.4	68.9	97.7
	Mean Rank	2.7	4.7	4.3	2.2	1.1
All cause death						
	SUCRA	13.5	24.6	60.2	90.3	61.4
	Mean Rank	4.5	4.0	2.6	1.4	2.5
Myocardial infarction						
	SUCRA	0.4	52.7	37.3	69.9	89.6
	Mean Rank	5	2.9	3.5	2.2	1.4
Stroke						
	SUCRA	37.0	52.8	49.8	40	70.5
	Mean Rank	3.5	2.9	3.0	3.4	2.2
Stent thrombosis						
	SUCRA	2.2	77.5	44.0	50.7	75.6
	Mean Rank	4.9	1.9	3.2	3.0	2.0

*SUCRA, surface under the cumulative ranking.

### 3.6 Loop-Specific Heterogeneity Test and Publication Bias Analysis

There is no obvious loop-specific heterogeneity in all sub-analyses in the included RCTs (**Supplementary Fig. 4**). We also performed funnel plot analyses, no publication bias of sub-analyses was observed (**Supplementary Fig. 5**).

## 4. Discussion

Our study is the first network meta-analysis to evaluate different antiplatelet therapies, especially DAPT de-escalation and potent P2Y12 inhibitor monotherapy, among ACS patients undergoing PCI. The paramount findings are as follows: (1). compared with conventional DAPT and potent P2Y12 inhibitor monotherapy, the DAPT de-escalation was associated with lower risks of net adverse clinical events in ACS patients; (2). compared with potent P2Y12 inhibitors-based DAPT, the net clinic benefit of DAPT de-escalation and monotherapy mainly was derived from a lower risk of bleeding, this advantage does not occur at the cost of an increase in MACEs. (3). ticagrelor or prasugrel-based DAPT was associated with a significant advantage over clopidogrel-based DAPT with lower ischemic events of myocardial infarction and stent thrombosis.

The long-term clinic benefits of patients with ACS are closely related to the types of antiplatelet interventions and implanted stents [22, 23]. Current guidelines recommend the use of a potent P2Y12 inhibitor (ticagrelor or prasugrel) combined with aspirin for antiplatelet therapy in ACS patients [2, 24]. However, the existing literature indicates the chronic treatment for 12 months of ticagrelor or other potent antiplatelet therapies after drug-eluting stents implantation was associated with increased bleeding risk [19, 25, 26, 27]. Recently released data suggested that lower-intensity antiplatelet agents were associated with a reduction of adverse outcomes for PCI-treated patients [28, 29]. The TWILIGHT trial compared the ticagrelor-based DAPT for 12 months and 3 months of DAPT plus 9 months of ticagrelor monotherapy, which demonstrated that ticagrelor monotherapy induced BARC bleeding or TIMI major bleeding events were significantly decreased over ticagrelor plus aspirin while no significant increase of MACEs was observed [9]. Given the bleeding events were significantly associated with a poor prognosis of death among ACS patients, antiplatelet therapies and timing of platelet inhibition have been the hotspot of clinical doctors [30].

Although 34.1%–44.4% of patients with acute myocardial infarction (AMI) in clinical practice switched from potent P2Y12 receptor inhibitors to clopidogrel-based antiplatelet therapy due to economic or pharmacological reasons, yet guidelines suggested that the clinical evidence for the conversion of different P2Y12 receptor inhibitors remains controversial [2, 19]. In the TOPIC trial, ACS patients treated with aspirin and newer P2Y12 inhibitors for 1 month were assigned to aspirin plus clopidogrel (DAPT de-escalation) or continued with the original therapy (control group), the results showed that there was no significant difference in ischemic events between two agents [7]. The net clinical benefit in the DAPT de-escalation was due to the reduction of bleeding events [7].

A recently released network meta-analysis (enrolled 15 RCTs with 55,798 patients) recommended DAPT de-escalation has superiority over conventional DAPT by comparing the clinical net benefits of ACS patients [31]. We conducted our network meta-analysis on the published large RCTs to provide clinical evidence for ACS patients by comparing the clinic outcomes of different antiplatelet therapies, especially DAPT de-escalation and potent P2Y12 monotherapy. We did a seminal work to suggest DAPT de-escalation was superior to potent P2Y12 monotherapy, followed by prasugrel plus aspirin, ticagrelor combined with aspirin, and clopidogrel plus aspirin in the net clinical benefit aspect of MACEs and bleeding events for patients with compelling indications.

The results of sub-analysis indicated that the net clinical benefits of DAPT de-escalation and potent P2Y12 inhibitor monotherapy primarily derived from the lower risk of major bleeding events and bleeding-induced adverse outcomes. Those results also revealed there might be a paradoxical finding that low-intensity antiplatelet therapies seemed to take slight advantages over potent DAPT therapies for reducing MACEs. Some potential hypotheses might be able to interpret the root of this finding. Since the risk of ischemic complications was widely accepted to be higher in the early phase of ACS or after PCI, the included RCTs indicated that patients of both monotherapy and DAPT de-escalation groups maintained prasugrel or ticagrelor-based DAPT for 1–3 months to avoid acute ischemic events, which may blunt the differences in MACEs between the standard DAPT, especially the clopidogrel-based DAPT group, and low-intensity antiplatelet strategies [10, 32]. Furthermore, the notably high rate of crossovers or nonadherence should be taken into consideration. For instance, in the TICO trial, 208 patients in the ticagrelor-based DAPT group changed their antiplatelet strategy for unplanned reasons, 83 patients switched to low-intensity DAPT or other strategies because of high bleeding risks, 112 patients discontinued the ticagrelor treatment because of side-effect of dyspnea [18]. In the GLOBAL LEADERS trial, the potential influence of nonadherence might be more significant. 766 patients in the low-intensity antiplatelet strategy group were not adherent to the original plan, because of the need for a more potent DAPT strategy, in addition, 442 patients suspended potent DAPT for medical reasons [17]. We doubt the crossovers or nonadherence of low-intensity antiplatelet and potent DAPT strategies may induce potential selection bias, which might counteract the slight advantages of low-intensity antiplatelet therapies in the MACEs.

We have to point out the understanding of the switch timing of the P2Y12 inhibitors wasn’t unified. We excluded the TROPICAL-ACS trial because of short DAPT treatment for 7 days, which didn’t match the inclusion criteria of the potent DAPT treatment for the acute phase of ACS should be at least 1 month [33]. Several studies suggested there might be larger response variability and high platelet reactivity after DAPT de-escalation from a potent P2Y12 inhibitor to clopidogrel, due to the peculiar bio-transfer process of the prodrug of clopidogrel [34, 35]. Hence, the TROPICAL-ACS trial aimed to investigate the effects of DAPT de-escalation from prasugrel to clopidogrel guided by platelet function testing, and the results suggested the DAPT de-escalation guided by genetic or platelet function testing was non-inferior to standard DAPT based on prasugrel [33]. Due to the scarcity of available studies and conclusive data, we failed to compare the effect between the genetic or platelet function testing unguided and guided DAPT de-escalation strategies. A meta-analysis (enrolled 5 RCTs with 10,779 patients) compared genetic or platelet function guided and unguided DAPT de-escalation with conventional DAPT based on potent P2Y12 inhibitors respectively, indicated that for ACS patients who underwent PCI, both unguided and guided DAPT de-escalation strategies were associated with lower risk of adverse endpoints of bleeding and ischemic events [36]. Furthermore, some scholars supported that guided selection of antiplatelet therapy, both guided DAPT escalation (switching from clopidogrel to prasugrel or ticagrelor) and de-escalation, can improve net clinic benefit in patients undergoing PCI. For genetic or platelet function testing guided DAPT escalation, it was proved to be associated with reductions in MACEs compared with standard DAPT therapy without the cost of an increase in bleeding events. It was demonstrated that the net clinic benefits mainly derive from fewer incidences of cardiovascular death and myocardial infarction [37]. As expected, guided DAPT de-escalation may induce reductions in bleeding events compared with standard therapy [37]. Another authoritative meta-analysis also supported the broader adoption of guided selections of P2Y12 inhibitors in patients with ACS by comparing guided antiplatelet therapy with conventional DAPT [38]. It is believed that compared with a routine selection of prasugrel, ticagrelor, or clopidogrel, a guided selection of P2Y12 inhibitor would be associated with the most optimal balance between safety and efficacy [38]. Since the absence of patient-level baseline data, those meta-analyses failed to conduct sub-group analyses on high-risk individuals, the effect of guided antiplatelet therapy remains for further research.

Some scholars believe East Asian people would be different in the risk of thrombosis and hemorrhagic events due to lower body mass and a higher risk of CYP2C19 loss-of-function compared to European and American Caucasian populations [39]. Low-dose ticagrelor or prasugrel has been determined to be superior to clopidogrel with a lower incidence of MACEs while not increasing the risk of bleeding among the Asian population, since low-dose potent P2Y12 inhibitors achieved low rates of high on-treatment platelet reactivity, which may imply a better antiplatelet effect [39, 40]. A recently released meta-analysis suggested the effect of DAPT de-escalation strategy could also be affected by different races, it reported that DAPT de-escalation was associated with a lower risk of bleeding events, which was only demonstrated in East Asian patients, and not in non-East Asian patients [41]. Since the TOPIC trial attributed net clinical benefit in the DAPT de-escalation to the reduction of bleeding events, we suspect DAPT de-escalation would be more effective in net clinical benefit among the Asian population [7].

Subgroup analysis suggested that prasugrel or ticagrelor plus aspirin were significantly associated with a lower risk of ischemic events of MI and ST than clopidogrel plus aspirin. Some researchers suggested the P2Y12 inhibitor monotherapy can’t reverse the diabetes-induced high risk of ischemic events by comparing it with different antiplatelet therapies in Korean patients with or without diabetes, which suggested that ACS patients, who are at high risk of ischemia need to maintain potent antiplatelet agents [42]. The TICO sub-study indicated that switching ticagrelor monotherapy after 3 months of DAPT can’t reduce ischemic events in patients with multivessel disease [18]. Due to the significant correlation between the hazards of diabetes, multivessel disease, and races, we believe that antiplatelet strategies for ACS people with high ischemic risk are still worthy of further exploration [43, 44].

## 5. Study Limitations

There were several limitations in our work: (1). Due to differences of primary endpoints among the references, bleeding evaluation criteria such as TIMI, PLATO and BARC bleeding cannot be converted to each other, which may affect the overall results. (2). Due to the limited studies of directly comparing different antiplatelet agents, the results of sub-analysis may be discordant with SUCRA value, more RCTs are still needed to analyze the clinic benefits of potential optimal antiplatelet therapies in various populations. (3). Due to the scarcity of available data on different races, we failed to analyze the effect of those antiplatelet strategies on the white, black, and Asian populations.

## 6. Conclusions

The network meta-analysis advised that DAPT de-escalation would reduce the net adverse clinical events, compared with potent P2Y12 inhibitor monotherapy, for ACS patients undergone PCI treatment. This study suggests that DAPT de-escalation may prevail over potent P2Y12 inhibitor monotherapy in ACS-PCI patients with a high risk of ischemia and bleeding.

## Abbreviations

DES, drug-eluting stent; DAPT, dual antiplatelet therapy; ACS, acute coronary syndrome; MI, myocardial infarction; MACE, major cardiovascular adverse event; PCI, percutaneous coronary intervention; RCT, randomized clinical trial; ST, stent thrombosis; TIMI, thrombolysis in myocardial infarction; BARC, bleeding academic research consortium; PLATO, platelet inhibition and patient outcomes; OR, odds ratio; CI, 95% confidence intervals; ROR, Relative Odds Ratio; SUCRA, surface under the cumulative ranking; ESC, European society of cardiology; EACTS, European association for cardio-thoracic surgery.

## Supplementary Material

Supplementary material associated with this article can be found, in the online version, at https://doi.org/10.31083/j.rcm2311360.
