# Supplementary material for: De-Escalation Dual Antiplatelet Therapy Prevail over Potent P2Y12 Inhibitor Monotherapy in Patients with Acute Coronary Syndrome Undergone Percutaneous Coronary Intervention: A Network Meta-Analysis
Source: Rev Cardiovasc Med. 2022 Oct 25;23(11):360. doi: 10.31083/j.rcm2311360 (PMC11269070; doi:10.31083/j.rcm2311360)
Supplement: Supplementary file 1 [file 2153-8174-23-11-360-s1.zip › 2153-8174-23-11-360-s1/Supplementary Fig. 3.pdf]

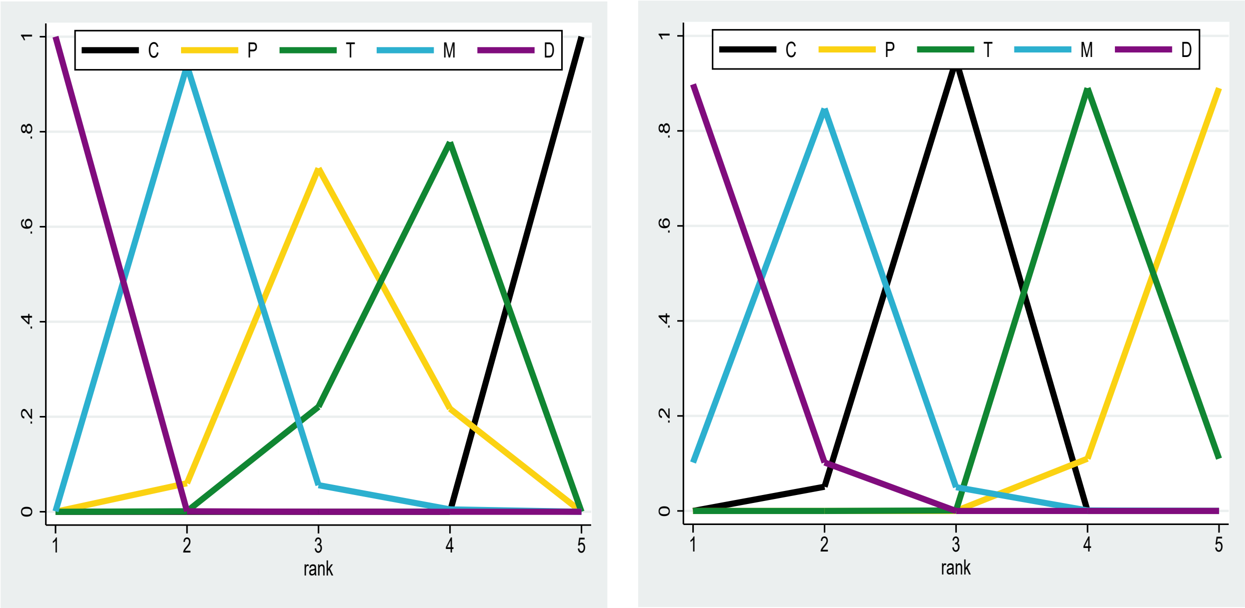

a.Cumulative probability rank plots for the primary efficacy outcomes

b.Cumulative probability rank plots for the primary safety outcomes

Appendix Fig.3 Cumulative probability rank plots of efficacy outcomes (a) and safety outcomes (b)
C=clopidogrel + aspirin; P=prasugrel + aspirin; T=ticagrelor + aspirin; D=de-escalation; M=P2Y12 Inhibitor monotherapy
