# Supplementary material for: De-Escalation Dual Antiplatelet Therapy Prevail over Potent P2Y12 Inhibitor Monotherapy in Patients with Acute Coronary Syndrome Undergone Percutaneous Coronary Intervention: A Network Meta-Analysis
Source: Rev Cardiovasc Med. 2022 Oct 25;23(11):360. doi: 10.31083/j.rcm2311360 (PMC11269070; doi:10.31083/j.rcm2311360)
Supplement: Supplementary file 1 [file 2153-8174-23-11-360-s1.zip › 2153-8174-23-11-360-s1/Supplementary Fig. 4.pdf]

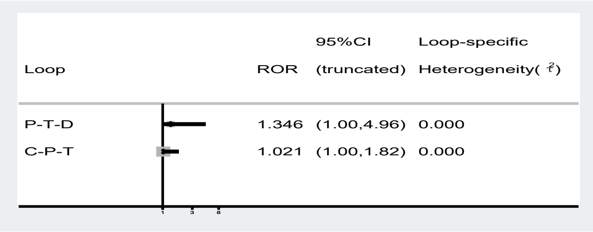

a.loop-specific heterogeneity test of all cause death

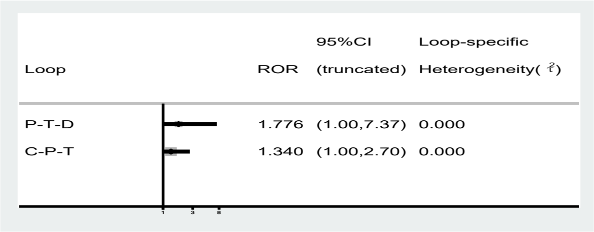

b.loop-specific heterogeneity test of MI

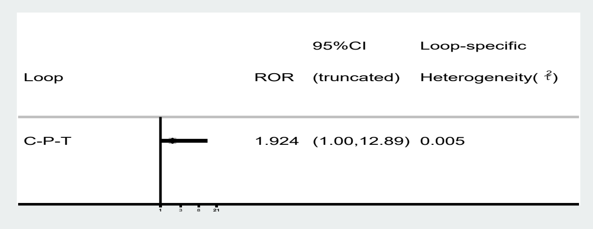

c.loop-specific heterogeneity test of primary efficacy outcomes of STEMI

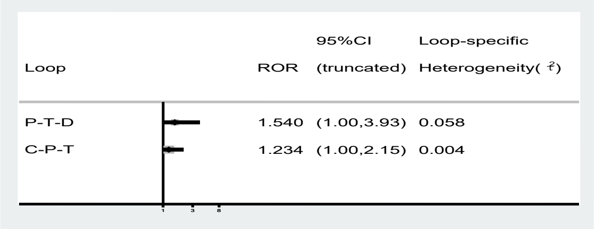

d.loop-specific heterogeneity test of primary efficacy outcomes

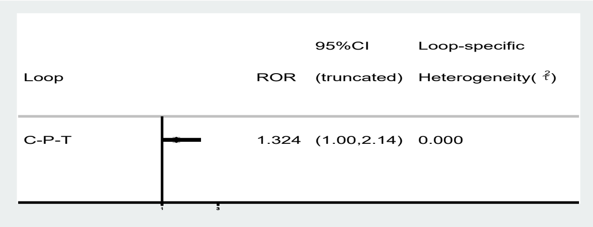

e.loop-specific heterogeneity test of primary safety outcomes of NSTEMI

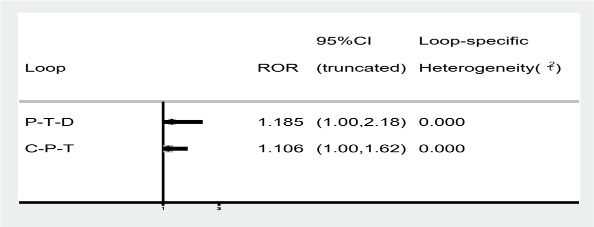

f.loop-specific heterogeneity test of primary safety outcomes

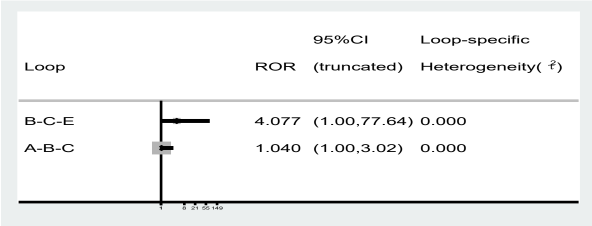

g.loop-specific heterogeneity test of ST

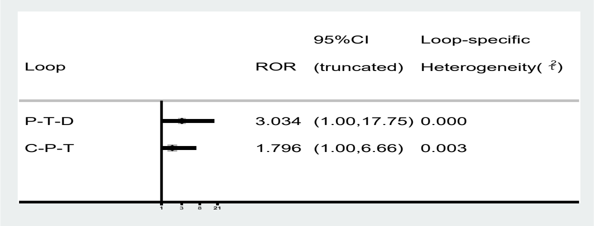

h.loop-specific heterogeneity test of stroke

Appendix Fig.4 loop-specific heterogeneity test
