# Supplementary material for: De-Escalation Dual Antiplatelet Therapy Prevail over Potent P2Y12 Inhibitor Monotherapy in Patients with Acute Coronary Syndrome Undergone Percutaneous Coronary Intervention: A Network Meta-Analysis
Source: Rev Cardiovasc Med. 2022 Oct 25;23(11):360. doi: 10.31083/j.rcm2311360 (PMC11269070; doi:10.31083/j.rcm2311360)
Supplement: Supplementary file 1 [file 2153-8174-23-11-360-s1.zip › 2153-8174-23-11-360-s1/Supplementary Fig. 5.pdf]

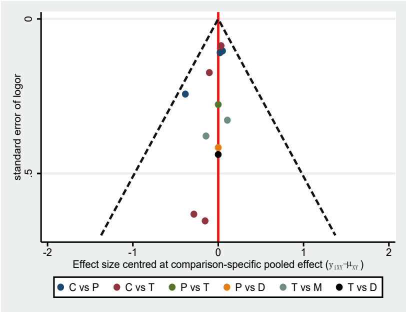

a.funnel plot analysis of all cause death

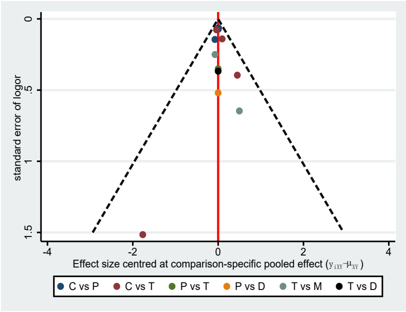

b.funnel plot analysis of MI

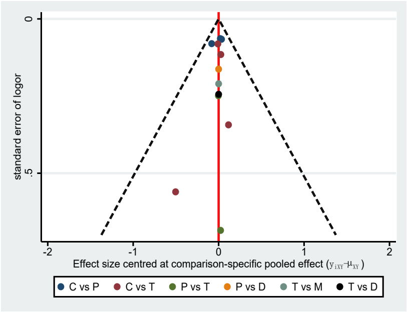

c.funnel plot analysis of primary efficacy of NSTEMI

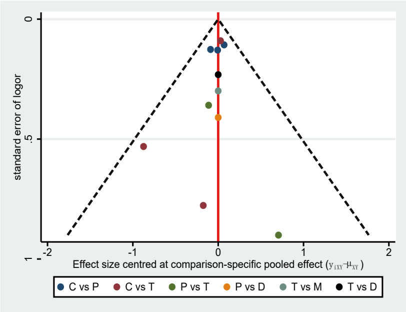

d.funnel plot analysis of primary efficacy of STEMI

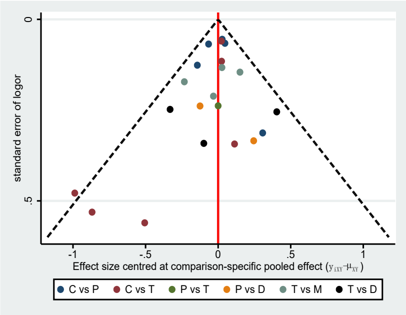

e.funnel plot analysis of primary efficacy outcomes

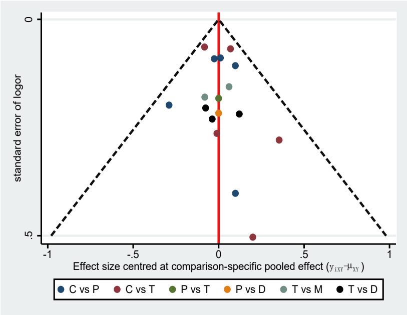

g.funnel plot analysis of primary safety outcomes

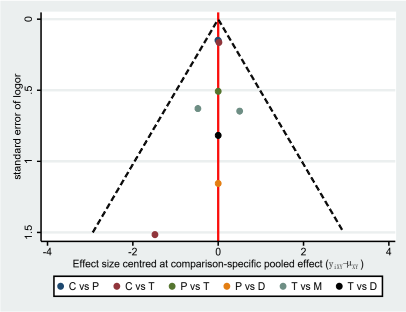

h.funnel plot analysis of ST

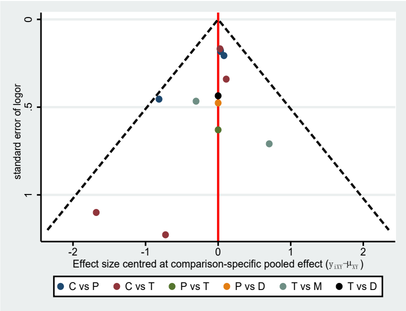

i.funnel plot analysis of stroke

Appendix Fig.5 Funnel plot analysis

C=clopidogrel + aspirin; P=prasugrel + aspirin; T=ticagrelor + aspirin; D=de-escalation;

M=P2Y12 inhibitor montherapy
