# Supplementary material for: De-Escalation Dual Antiplatelet Therapy Prevail over Potent P2Y12 Inhibitor Monotherapy in Patients with Acute Coronary Syndrome Undergone Percutaneous Coronary Intervention: A Network Meta-Analysis
Source: Rev Cardiovasc Med. 2022 Oct 25;23(11):360. doi: 10.31083/j.rcm2311360 (PMC11269070; doi:10.31083/j.rcm2311360)
Supplement: Supplementary file 1 [file 2153-8174-23-11-360-s1.zip › 2153-8174-23-11-360-s1/Supplementary Table 1.docx]

Supplementary Table 1. League table with respect to sub-analyses outcomes

| **MACE of STEMI** | | | |  | |  | | |  | |
| --- | --- | --- | --- | --- | --- | --- | --- | --- | --- | --- |
| D | 1.27 (0.63,2.58) | | | 1.55 (1.01,2.36) | | 1.77 (1.19,2.63) | | | 2.13 (1.41,3.23) | |
| 0.79 (0.39,1.60) | M | | | 1.22 (0.65,2.26) | | 1.39 (0.77,2.50) | | | 1.68 (0.91,3.08) | |
| 0.65 (0.42,0.99) | 0.82 (0.44,1.53) | | | P | | 1.14 (0.93,1.40) | | | 1.38 (1.21,1.57) | |
| 0.57 (0.38,0.84) | 0.72 (0.40,1.29) | | | 0.87 (0.71,1.07) | | T | | | 1.21 (1.02,1.42) | |
| 0.47 (0.31,0.71) | 0.60 (0.32,1.10) | | | 0.73 (0.64,0.83) | | 0.83 (0.70,0.98) | | | C | |
| **MACE of NSTEMI&UA** | | | |  | |  | | |  | |
| D | 0.98 (0.59,1.61) | | | 1.57 (1.20,2.05) | | 1.58 (1.19,2.10) | | | 1.99 (1.51,2.61) | |
| 1.02 (0.62,1.69) | M | | | 1.60 (1.04,2.48) | | 1.62 (1.07,2.44) | | | 2.03 (1.32,3.12) | |
| 0.64 (0.49,0.83) | 0.62 (0.40,0.96) | | | P | | 1.01 (0.88,1.16) | | | 1.27 (1.17,1.37) | |
| 0.63 (0.48,0.84) | 0.62 (0.41,0.93) | | | 0.99 (0.86,1.14) | | T | | | 1.26 (1.12,1.42) | |
| 0.50 (0.38,0.66) | 0.49 (0.32,0.76) | | | 0.79 (0.73,0.85) | | 0.80 (0.71,0.90) | | | C | |
| **All cause death** | | | |  | |  | | |  | |
| M | 1.61 (0.99,2.61) | | | 1.53 (0.71,3.31) | | 1.89 (1.12,3.19) | | | 2.09 (1.26,3.47) | |
| 0.62 (0.38,1.01) | T | | | 0.95 (0.52,1.74) | | 1.18 (0.98,1.43) | | | 1.30 (1.13,1.50) | |
| 0.65 (0.30,1.41) | 1.05 (0.58,1.91) | | | D | | 1.24 (0.68,2.25) | | | 1.37 (0.75,2.49) | |
| 0.53 (0.31,0.89) | 0.85 (0.70,1.02) | | | 0.81 (0.44,1.47) | | P | | | 1.10 (0.96,1.26) | |
| 0.48 (0.29,0.79) | 0.77 (0.66,0.89) | | | 0.73 (0.40,1.33) | | 0.91 (0.79,1.04) | | | C | |
| **Myocardial infarction** | | | | |  | | |  | | |
| D | 1.39 (0.77,2.52) | | | 1.39 (0.66,2.94) | | 1.52 (0.84,2.75) | | | 1.86 (1.03,3.37) | |
| 0.72 (0.40,1.30) | P | | | 1.00 (0.62,1.62) | | 1.09 (0.95,1.26) | | | 1.34 (1.25,1.44) | |
| 0.72 (0.34,1.51) | 1.00 (0.62,1.61) | | | M | | 1.09 (0.69,1.73) | | | 1.34 (0.83,2.15) | |
| 0.66 (0.36,1.18) | 0.91 (0.79,1.06) | | | 0.91 (0.58,1.45) | | T | | | 1.22 (1.08,1.39) | |
| 0.54 (0.30,0.97) | 0.75 (0.70,0.80) | | | 0.75 (0.47,1.20) | | 0.82 (0.72,0.93) | | | C | |
| **Stroke** | | | |  | |  | | |  | |
| D | 1.07 (0.55,2.06) | | | 1.09 (0.56,2.09) | | 1.12 (0.41,3.05) | | | 1.14 (0.60,2.18) | |
| 0.94 (0.49,1.80) | P | | | 1.02 (0.83,1.24) | | 1.05 (0.46,2.41) | | | 1.07 (0.77,1.48) | |
| 0.92 (0.48,1.77) | 0.98 (0.80,1.20) | | | C | | 1.03 (0.46,2.32) | | | 1.05 (0.79,1.38) | |
| 0.89 (0.33,2.43) | 0.95 (0.41,2.19) | | | 0.97 (0.43,2.18) | | M | | | 1.01 (0.47,2.18) | |
| 0.88 (0.46,1.68) | 0.94 (0.67,1.30) | | | 0.95 (0.72,1.26) | | 0.99 (0.46,2.11) | | | T | |
| **Stent thrombosis** | | | | |  | | |  | | |
| D | | 1.23 (0.32,4.63) | 1.43 (0.29,6.95) | | | | 1.57 (0.42,5.83) | | | 2.56 (0.68,9.65) |
| 0.82 (0.22,3.08) | P | | | 1.16 (0.45,3.02) | | 1.28 (0.90,1.82) | | | 2.09 (1.71,2.57) | |
| 0.70 (0.14,3.41) | 0.86 (0.33,2.22) | | | M | | 1.10 (0.45,2.66) | | | 1.80 (0.71,4.57) | |
| 0.64 (0.17,2.37) | 0.78 (0.55,1.11) | | | 0.91 (0.38,2.20) | | T | | | 1.63 (1.21,2.21) | |
| 0.39 (0.10,1.47) | 0.48 (0.39,0.59) | | | 0.56 (0.22,1.42) | | 0.61 (0.45,0.83) | | | C | |

*Values are expressed as OR (95% Confidence Intervals).

C=clopidogrel + aspirin; P=prasugrel + aspirin; T=ticagrelor + aspirin; M=P2Y12 Inhibitor monotherapy; D=de-escalation.
