# Supplementary figures and images for: De-Escalation Dual Antiplatelet Therapy Prevail over Potent P2Y12 Inhibitor Monotherapy in Patients with Acute Coronary Syndrome Undergone Percutaneous Coronary Intervention: A Network Meta-Analysis
Source: Rev Cardiovasc Med. 2022 Oct 25;23(11):360. doi: 10.31083/j.rcm2311360 (PMC11269070; doi:10.31083/j.rcm2311360)

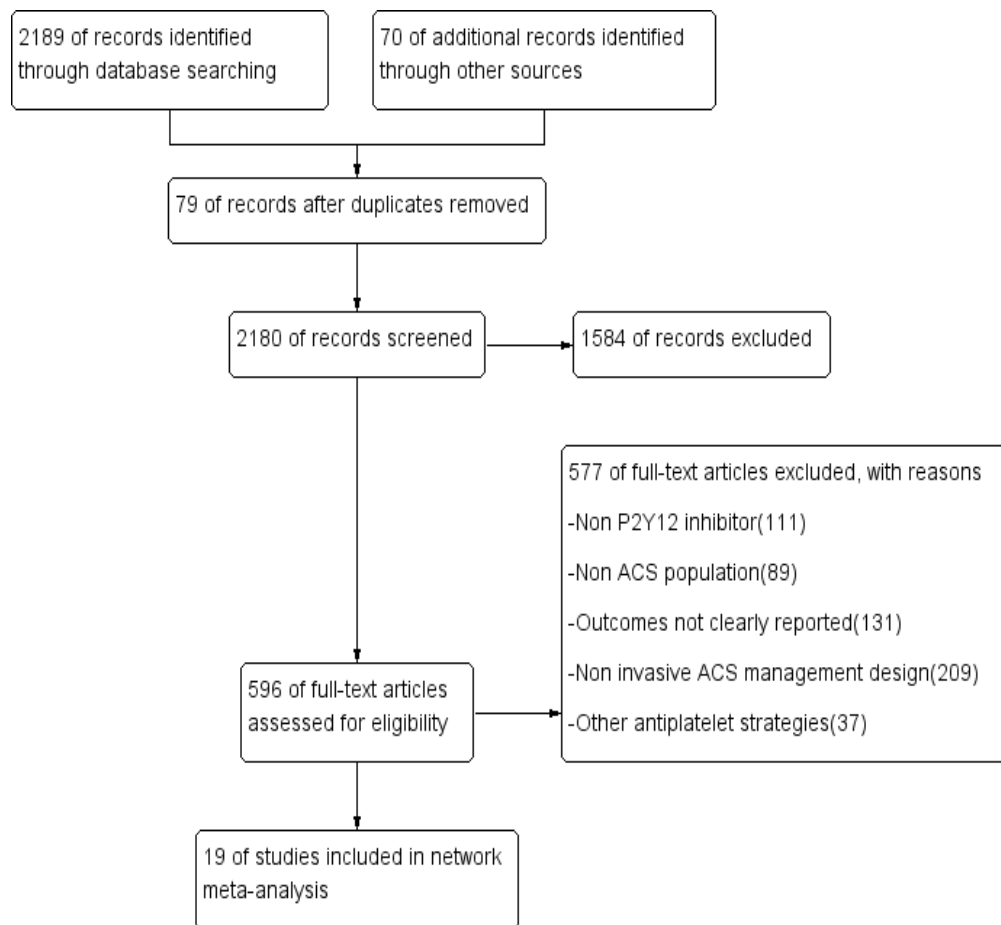

Appendix Fig.1 Flowchart of search strategy and article selection

Supplement: Supplementary file 1 [file 2153-8174-23-11-360-s1.zip › 2153-8174-23-11-360-s1/Supplementary Fig. 1.pdf]

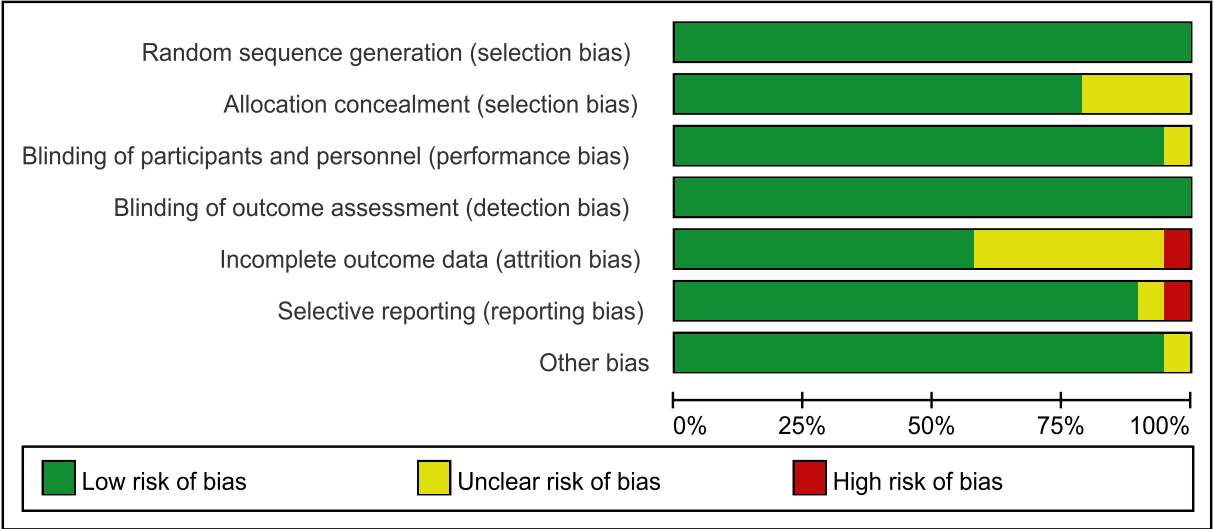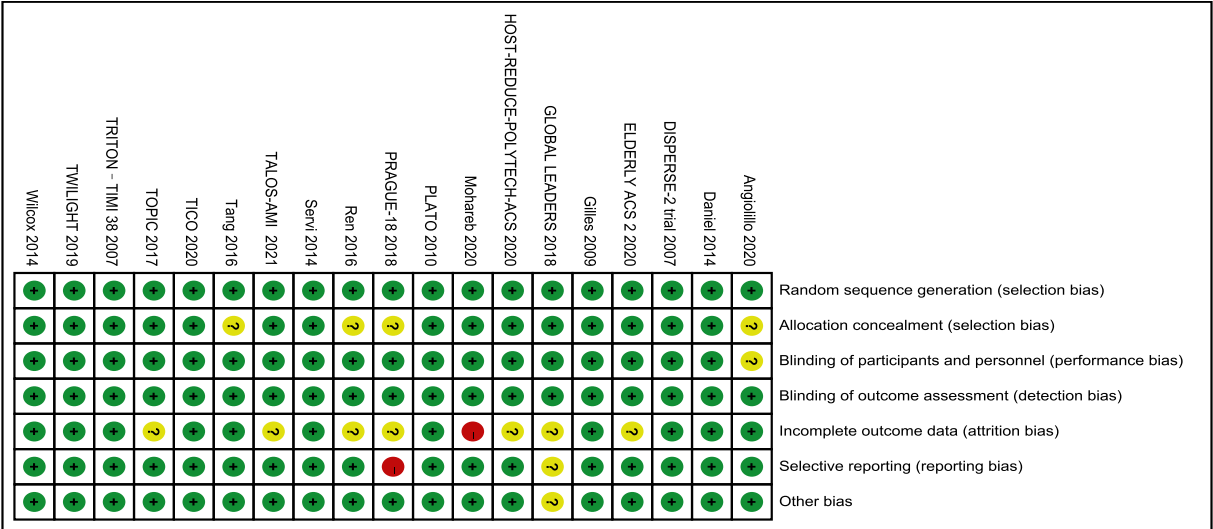

Appendix Fig.2 Risk of bias

Supplement: Supplementary file 1 [file 2153-8174-23-11-360-s1.zip › 2153-8174-23-11-360-s1/Supplementary Fig. 2.pdf]
